# DNA Methyltransferase 3b in Myeloid Cells Does Not Affect the Acute Immune Response in the Airways during *Pseudomonas* Pneumonia

**DOI:** 10.3390/cells11050787

**Published:** 2022-02-24

**Authors:** Wanhai Qin, Xanthe Brands, Cornelis van’t Veer, Alex F. de Vos, Brendon P. Scicluna, Tom van der Poll

**Affiliations:** 1 Center of Experimental & Molecular Medicine, Amsterdam University Medical Centers, Location Academic Medical Center, University of Amsterdam, 1105 AZ Amsterdam, The Netherlands; x.brands@amsterdamumc.nl (X.B.); c.vantveer@amsterdamumc.nl (C.v.V.); a.f.devos@amsterdamumc.nl (A.F.d.V.); b.scicluna@amsterdamumc.nl (B.P.S.); t.vanderpoll@amsterdamumc.nl (T.v.d.P.); 2 Department of Clinical Epidemiology, Biostatistics, and Bioinformatics, Amsterdam University Medical Centers, Location Academic Medical Center, University of Amsterdam, 1105 AZ Amsterdam, The Netherlands; 3 Division of Infectious Diseases, Amsterdam University Medical Centers, Location Academic Medical Center, University of Amsterdam, 1105 AZ Amsterdam, The Netherlands

**Keywords:** Dnmt3b, neutrophils, *P. aeruginosa*, pneumonia

## Abstract

DNA methyltransferase 3b (Dnmt3b) has been suggested to play a role in the host immune response during bacterial infection. Neutrophils and other myeloid cells are crucial for lung defense against *Pseudomonas (P.) aeruginosa* infection. This study aimed to investigate the role of Dnmt3b in neutrophils and myeloid cells during acute pneumonia caused by *P. aeruginosa*. Neutrophil-specific (*Dnmt3b^fl/fl^Mrp8^Cre^*) or myeloid cell-specific (*Dnmt3b^fl/fl^LysM^Cre^*) Dnmt3b-deficient mice and littermate control mice were infected with *P. aeruginosa* PAK via the airways. Bacteria burdens, neutrophil recruitment, and activation (CD11b expression, myeloperoxidase, and elastase levels), interleukin (IL)-1β, IL-6, and tumor necrosis factor (TNF) were measured in bronchoalveolar lavage fluid (BALF) at 6 and 24 h after infection. Our data showed that the bacterial loads and neutrophil recruitment and activation did not differ in BALF obtained from neutrophil-specific Dnmt3b-deficient and control mice, whilst BALF IL-6 and TNF levels were lower in the former group at 24 but not at 6 h after infection. None of the host response parameters measured differed between myeloid cell-specific Dnmt3b-deficient and control mice. In conclusion, dnmt3b deficiency in neutrophils or myeloid cells does not affect acute immune responses in the airways during *Pseudomonas* pneumonia.

## 1. Introduction

Accumulating evidence indicates that DNA methylation is involved in the regulation of host defense in bacterial infection [1]. Neonatal and adult patients with bacterial sepsis, as compared with control subjects, showed altered DNA methylation levels in blood leukocytes in genomic regions involved in immune responses [2,3]. Furthermore, the sepsis-associated aberrant DNA methylome is frequently related to dysregulated inflammatory responses and organ dysfunction [2,4].

DNA methylation is regulated by DNA methyltransferases (Dnmt’s), of which Dnmt3a and Dnmt3b mediate de novo methylation. Dnmt3b plays a major role in establishing DNA methylation patterns and, as a consequence, in gene expression [1]. Mutations in the human *DNMT3B* gene cause the immunodeficiency-centromeric instability-facial anomalies (ICF) syndrome [5], characterized by altered epigenetic modifications and expression of genes involved in the development and immune function [6]. In experimental sepsis in mice, inhibition of DNA methylation by 5-aza-2′-deoxycytidine, which targets Dnmt3a and Dnmt3b [7], improved survival by impeding NF-κB pathway activation [2]. Additional investigations have pointed to the role of Dnmt3b in the host response during infection. Patients with ICF syndrome normally present recurrent [8], and oftentimes severe, infections [9]. Exome sequencing showed an association of variants of *DNMT3B* with community-acquired *P. aeruginosa* infection in children [10]. We previously reported on the role of Dnmt3b in respiratory epithelial cells in *Pseudomonas* pneumonia, showing that targeted deletion of *Dnmt3b* in the bronchial epithelium-enhanced host antibacterial defense in the airways by promoting CXCL1 production and subsequent recruitment of neutrophils [11]. The role of Dntm3b partially depended on the presence of flagellin [11], an important virulence factor and Toll-like receptor 5 ligand expressed by *Pseudomonas* [11,12]. Epigenetic regulation of macrophage polarization by Dnmt3b has also been documented [13]. Together, these data led us to hypothesize that Dnmt3b in myeloid cells is involved in the host immune response during *Pseudomonas* pneumonia. To test this hypothesis, we generated neutrophil-specific or myeloid cell-specific Dnmt3b-deficient mice and infected these and littermate control mice with viable *P. aeruginosa* via the airways.

## 2. Materials and Methods

### 2.1. Animals

Homozygous *Dnmt3b^fl/fl^* mice [14] were crossed with *Mrp8^Cre^* mice (021614; Jackson Laboratory) [15] or *LysM^Cre^* mice [16] (Jackson Laboratory, Bar Harbor, ME) to generate neutrophil cell-specific Dnmt3b-deficient (*Dnmt3b^fl/fl^Mrp8^Cre^*) mice or myeloid cell-specific Dnmt3b-deficient (*Dnmt3b^fl/fl^ LysM^Cre^*) mice, respectively. Their corresponding *Dnmt3b^fl/fl^* Cre-negative littermates (*Dnmt3b^fl/fl^* mice) were used as controls in all experiments. All genetically modified mice were backcrossed at least eight times to a C57Bl/6 background, and age and sex matched when used in experiments. Mice were used at 8–12 weeks of age. All experiments were approved by the Institutional Animal Care and Use Committee of the University of Amsterdam.

### 2.2. Quantitative Reverse Transcription PCR (qRT-PCR)

Alveolar macrophages, peritoneal macrophages, and bone marrow-derived macrophages from *Dnmt3b^fl/fl^ LysM^Cre^* and *Dnmt3b^fl/fl^* were prepared as previously described [17]. Neutrophils were purified from the bone marrow of *Dnmt3b^fl/fl^ Mrp8^Cre^* and *Dnmt3b^fl/fl^* mice using Anti-Ly-6G MicroBead Kit (Miltenyi Biotec; Bergisch Gladbach, Germany) according to manufacturer’s instructions. RNA was isolated from these cells, and the expression of *Dnmt3a* and *Dnmt3b* was measured by qRT-PCR as described before [17]. The gene-specific primers used were, 5′-CTGAGTTTGACGCTTGGCGG-3′ and 5′-AGTCCACCTCCTCACAGGGT-3′ for *Dnmt3a*; 5′-GTGTGGGGAAAGATCAAGGG-3′ and 5′-AACTTGCCATCACCAAACCA-3′ for *Dnmt3b*; 5′-AGTCAAGGGCATATCCAACA-3′ and 5′-CAGCCCCAAAATGGTTAAGGT-3′ for *Hprt*.

### 2.3. Induction of Pneumonia and Sampling of Organs

Pneumonia was induced by intranasal inoculation with 5 × 10^6^ CFU *P. aeruginosa* PAK or 5 × 10^6^ CFU flagellin-deficient PAK (PAKflic) as described [12,18]. Bronchoalveolar lavage fluid (BALF) was collected at 6 or 24 h after infection. All the procedures were performed as described [12,18].

### 2.4. Assays

Interleukin (IL)-1β, IL-6, tumor necrosis factor (TNF), myeloperoxidase (MPO), and elastase were measured by enzyme-linked immunosorbent assays (ELISA; RnD Systems, Minneapolis, MN) according to manufacturer’s instructions.

### 2.5. Flow Cytometry

Flow cytometry was done on FACS Calibur (Becton Dickinson, Franklin Lakes, NJ, USA) after BAL cells were stained with fixable viability dye eFluor 780, rat anti mouse-CD45 PE-eFluor610 (30-F11), rat anti-mouse CD11b PE-Cy7 (clone M1/70), rat anti-mouse Siglec-F Alexa Fluor 647 (clone E50-2440), rat anti-mouse Ly-6C Alexa Fluor 700 (clone AL-21) (all from BD Biosciences), and rat anti-mouse Ly-6G FITC (clone 1A8; Biolegend, San Diego, CA). Neutrophils were identified as CD45+/Siglec-F-/CD11b+/Ly6C+/Ly6G+ cells. Data were analyzed using FlowJo software (Becton Dickinson) as described [19].

### 2.6. Statistical Analysis

Non-parametric variables were analyzed using the Mann–Whitney U test. Analysis was done using GraphPad Prism version 8 (Graphpad Software, San Diego, CA, USA). Statistical significance is shown as * *p* < 0.05.

## 3. Results

**Figure 1:**
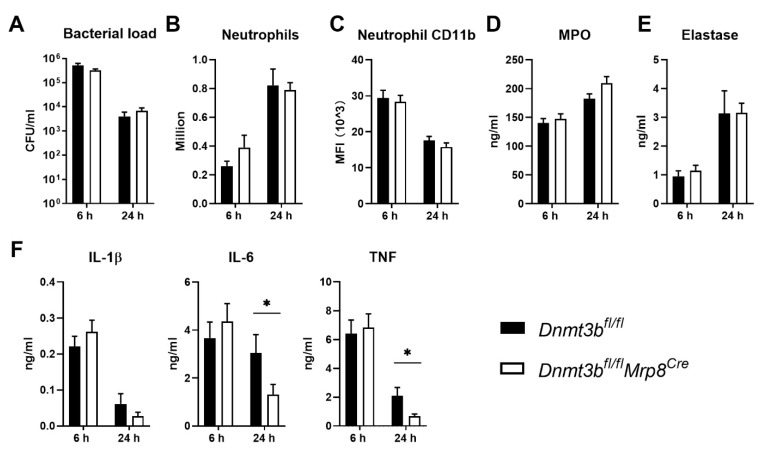
Neutrophil-specific Dnmt3b deficiency does not affect the acute host response during *Pseudomonas aeruginosa* pneumonia. Neutrophil-specific Dnmt3b-deficient mice (*Dnmt3b^fl/fl^Mrp8^Cre^*) and littermate control (*Dnmt3b^fl/fl^*) mice were infected with viable PAK (5 × 10^6^ CFU) via the airways and euthanized 6 or 24 h after infection. (**A**) Bacterial counts (colony forming units, CFU), (**B**) neutrophil numbers, (**C**) neutrophil CD11b expression, (**D**) myeloperoxidase (MPO), (**E**) elastase, (**F**) IL-β, IL-6, TNF levels, all in bronchoalveolar lavage fluid (BALF). Data are shown as bar graphs with mean + SEM, *n* = 8. * *p* < 0.05.

Dnmt3b is a de novo methyltransferase that has been widely studied as a regulator of DNA methylation [20]. More recent studies implicated Dnmt3b in the regulation of the host immune response during bacterial infection in general and *P. aeruginosa* infection in particular [2,10,11]. Neutrophils are the first cells recruited upon infection of the airways by *P. aeruginosa,* where they play a crucial role in host defense against this bacterium [21,22]. To determine the role of Dnmt3b in neutrophils during *Pseudomonas* pneumonia, we crossed *Mrp8^Cre^* mice, which specifically targets floxed gene segments in neutrophils [23] with *Dnmt3b^fl/fl^* mice to generate neutrophil-specific *Dnmt3b* knockout (*Dnmt3b^fl/fl^Mrp8^Cre^*) and littermate control (*Dnmt3b^fl/fl^*) mice, and infected these with PAK via the airways. Dnmt3b but not Dnmt3a mRNA level in neutrophils of naïve *Dnmt3b^fl/fl^Mrp8^Cre^* mice was significantly lower than that of littermate control mice (Figure S1), confirming the neutrophil-specific knockout of Dnmt3b in *Dnmt3b^fl/fl^Mrp8^Cre^* mice. Bacterial loads and neutrophil numbers in BALF were similar in both mouse strains at 6 and 24 h after infection (Figure 1A,B). Likewise, neutrophil activation, determined by quantification of cell surface CD11b expression and by measuring the neutrophil degranulation products MPO and elastase in BALF was comparable in *Dnmt3b^fl/fl^Mrp8^Cre^* and control mice at both time points after *Pseudomonas* infection (Figure 1C–E). BALF levels of the proinflammatory cytokines IL-1β, IL-6, and TNF were similar in both mouse strains at 6 h after infection. At 24 h, cytokine concentrations had decreased in both groups but to a greater extent in *Dnmt3b^fl/fl^Mrp8^Cre^* mice than in control mice (significantly so for IL-6 and TNF; Figure 1F). Taken together, these data indicate that neutrophil Dnmt3b does not affect key immune responses or antibacterial defense during *P. aeruginosa* pneumonia.

**Figure 2:**
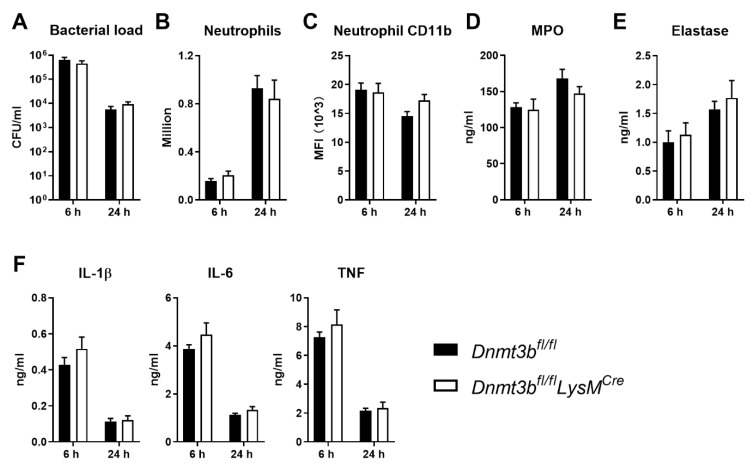
Myeloid cell Dnmt3b deficiency does not affect the acute host response during *Pseudomonas aeruginosa* pneumonia. Myeloid cell-specific Dnmt3b-deficient mice (*Dnmt3b^fl/fl^LysM^Cre^*) and littermate control **(***Dnmt3b^fl/fl^*) mice were infected with viable PAK (5 × 10^6^ CFU) via the airways and euthanized 6 or 24 h after infection. (**A**) Bacterial counts (colony forming units, CFU), (**B**) neutrophil numbers, (**C**) neutrophil CD11b expression, (**D**) myeloperoxidase (MPO), (**E**) elastase, (**F**) IL-β, IL-6 and TNF levels, all in bronchoalveolar lavage fluid (BALF). Data are shown as bar graphs with mean + SEM, *n* = 8. Differences between groups were not significant.

Considering that Dnmt3b affects macrophages polarization [13], we next induced *Pseudomonas* pneumonia in *Dnmt3b^fl/fl^ LysM^Cre^* mice (in which *Dnmt3b* is predominately deleted in macrophages as confirmed at mRNA levels (Figure S2)), and partially in neutrophils and monocytes [23]) and control mice. Both at 6 and 24 h after infection, bacterial burdens, neutrophil recruitment, neutrophil activation (CD11b expression, MPO and elastase production in the BALF), and pro-inflammatory cytokines (IL-1β, IL-6 and TNF) levels were similar in both mouse strains (Figure 2A–F). Flagellin is a crucial virulence factor expressed by *P. aeruginosa* that potentially activates the respiratory epithelium [12]. We speculated that a possible role of myeloid Dntm3b could be obscured by a dominant effect of flagellin on epithelial cells during infection of the airways with *P. aeruginosa*. To test this possibility, we infected *Dnmt3b^fl/fl^ LysM^Cre^* and control mice with flagellin-deficient PAK (PAKflic). Six hours after infection with PAKflic bacterial burdens, neutrophil recruitment, and activation, and cytokine concentrations in BALF were similar in both mouse strains (Figure S3). Collectively, these data suggest that Dnmt3b in myeloid cells does not play a significant role in the host response during pneumonia caused by acute *P. aeruginosa* infection.

## 4. Discussion

*P. aeruginosa* is one of the most frequent causative pathogens in nosocomial pneumonia [24]. Both neutrophils and macrophages play vital roles in host defense against pulmonary *P. aeruginosa* infection [24]. Whilst genetic studies in humans have implicated Dnmt3b in host defense to *P. aeruginosa* infection [8,9,10], the data presented here strongly argue against the role of myeloid Dnmt3b in *Pseudomonas* pneumonia. Previously, it was demonstrated that knock-down of Dnmt3b in macrophages resulted in decreased expression and secretion of pro-inflammatory cytokines in response to in vitro stimulation with lipopolysaccharide [13], a major component of the outer membrane of gram-negative bacteria like *P. aeruginosa*. Opposite to these results, we show here that the absence of Dnmt3b in macrophages does not impact on cytokine production during *Pseudomonas* infection in vivo. Interestingly, we have recently shown that Dnmt3b in bronchial epithelial cells impairs the clearance of *P. aeruginosa* from the airways by a mechanism that involves inhibition of mucosal responses to bacterial flagellin by an effect on epithelial cell DNA methylation [11]. Further research into the cell-specific role of Dnmt3b in host defense to infection is, therefore, warranted.

In conclusion, the results of the present study indicate that Dnmt3b in myeloid cells is dispensable for host defense against pulmonary *P. aeruginosa* infection and does not regulate the acute immune response in the airways during *Pseudomonas*-induced pneumonia. These findings suggest that Dnmt3b functions in a cell-specific way during the host response in pneumonia caused by *P. aeruginosa*.

## Data Availability

All data generated or analyzed in this study are included in the manuscript.

## Supplementary Materials

The following supporting information can be downloaded at: https://www.mdpi.com/article/10.3390/cells11050787/s1, Figure S1: Dnmt3b and Dnmt3a mRNA levels in neutrophils of *Dnmt3b^fl/fl^Mrp8^Cre^* mice and their littermate control mice (*Dnmt3b^fl/fl^*); Figure S2: tDnmt3b (A) and Dnmt3a mRNA levels (B) in alveolar macrophages (AMs), peritoneal macrophages (PM) and bone marrow-derived macrophages (BMDMs) of *Dnmt3b^fl/fl^LysM^Cre^* mice and their littermate control mice (*Dnmt3b^fl/fl^*); Figure S3: Myeloid cell Dnmt3b deficiency does not affect the host response during acute pneumonia caused by flagellin deficient *Pseudomonas aeruginosa*.
