# Supplementary material for: DNA Methyltransferase 3b in Myeloid Cells Does Not Affect the Acute Immune Response in the Airways during Pseudomonas Pneumonia
Source: Cells. 2022 Feb 24;11(5):787. doi: 10.3390/cells11050787 (PMC8909799; doi:10.3390/cells11050787)

Supplementary Document

Supplementary Figure S1. Dnmt3b and Dnmt3a mRNA levels in neutrophils of *Dnmt3b$^{fl/fl}$Mrp8^{Cre}* mice and their littermate control mice (*Dnmt3b$^{fl/fl}$*). Data is shown as bar graphs with mean + SEM, n = 6. *p < 0.05

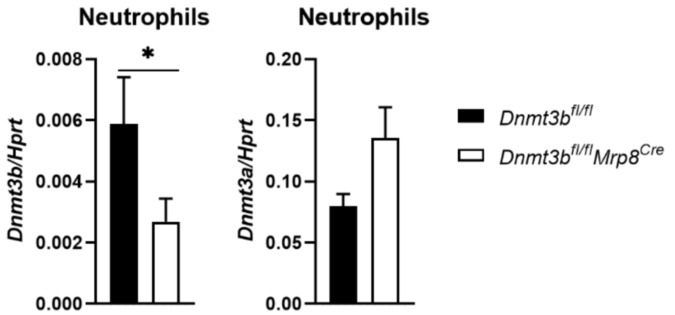

Supplementary Figure S2. Dnmt3b (A) and Dnmt3a mRNA levels (B) in alveolar macrophages (AMs), peritoneal macrophages (PM) and bone marrow-derived macrophages (BMDMs) of *Dnmt3b$^{fl/fl}$LysM^{Cre}* mice and their littermate control mice (*Dnmt3b$^{fl/fl}$*). Data is shown as bar graphs with mean + SEM, n = 6. *p < 0.05

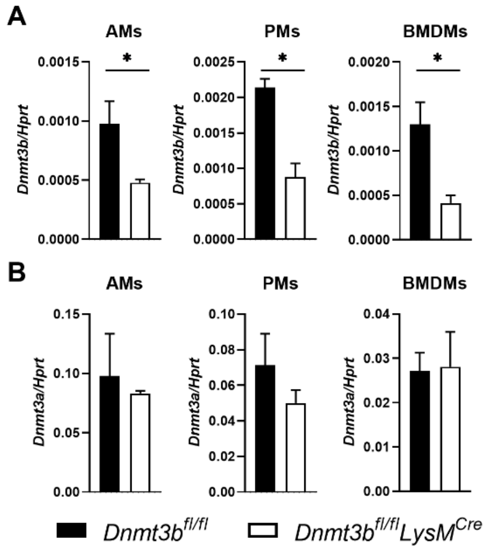

Supplementary Figure S3. Myeloid cell Dnmt3b deficiency does not affect the host response during acute pneumonia caused by flagellin deficient *Pseudomonas aeruginosa*. Myeloid cell specific Dnmt3b deficient mice (*Dnmt3b*$^{fl/fl}$*LysM*^{Cre}) and littermate controls (*Dnmt3b*$^{fl/fl}$) were infected with viable flagellin deficient PAK (5 x 10^6 CFU) via the airways. 6 h after infection, (A) bacterial counts (colony forming units, CFU), (B) neutrophil numbers, (C) neutrophil CD11b expression, (D) myeloperoxidase (MPO), (E) elastase, (F) IL-β, IL-6 and TNF levels in bronchoalveolar lavage fluid (BALF) were determined. Data is shown as bar graphs with mean + SEM, n = 8.

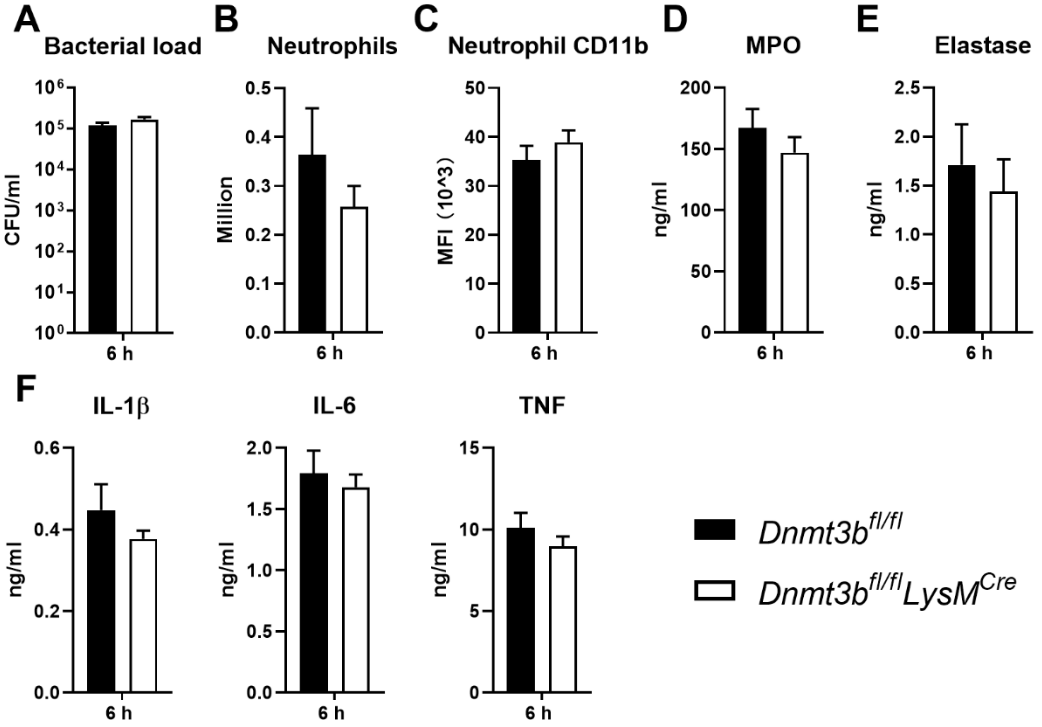

Supplement: Supplementary file 1 [file cells-11-00787-s001.zip › cells-1596777-supplementary.pdf]
